# Comparative Analysis of Complete Chloroplast Genome Sequences of Wild and Cultivated *Bougainvillea* (Nyctaginaceae)

**DOI:** 10.3390/plants9121671

**Published:** 2020-11-28

**Authors:** Mary Ann C. Bautista, Yan Zheng, Zhangli Hu, Yunfei Deng, Tao Chen

**Affiliations:** 1 South China Botanical Garden, Chinese Academy of Sciences, Guangzhou 510650, China; bautista.maryann@gmail.com; 2 Fairy Lake Botanical Garden, Chinese Academy of Sciences, Shenzhen 518004, China; yanzheng@szbg.ac.cn; 3 Graduate School, University of Chinese Academy of Sciences, Beijing 100049, China; 4 School of Life Sciences and Oceanology, Shenzhen University, Shenzhen 518060, China; huzl@szu.edu.cn

**Keywords:** *Bougainvillea*, Nyctaginaceae, chloroplast genome, phylogeny

## Abstract

*Bougainvillea* (Nyctaginaceae) is a popular ornamental plant group primarily grown for its striking colorful bracts. However, despite its established horticultural value, limited genomic resources and molecular studies have been reported for this genus. Thus, to address this existing gap, complete chloroplast genomes of four species (*Bougainvillea glabra, Bougainvillea peruviana, Bougainvillea pachyphylla, Bougainvillea praecox*) and one *Bougainvillea* cultivar were sequenced and characterized. The *Bougainvillea* cp genomes range from 153,966 bp to 154,541 bp in length, comprising a large single-copy region (85,159 bp–85,708 bp) and a small single-copy region (18,014 bp–18,078 bp) separated by a pair of inverted repeats (25,377–25,427 bp). All sequenced plastomes have 131 annotated genes, including 86 protein-coding, eight rRNA, and 37 tRNA genes. These five newly sequenced *Bougainvillea* cp genomes were compared to the *Bougainvillea spectabilis* cp genome deposited in GeBank. The results showed that all cp genomes have highly similar structures, contents, and organization. They all exhibit quadripartite structures and all have the same numbers of genes and introns. Codon usage, RNA editing sites, and repeat analyses also revealed highly similar results for the six cp genomes. The amino acid leucine has the highest proportion and almost all favored synonymous codons have either an A or U ending. Likewise, out of the 42 predicted RNA sites, most conversions were from serine (S) to leucine (L). The majority of the simple sequence repeats detected were A/T mononucleotides, making the cp genomes A/T-rich. The contractions and expansions of the IR boundaries were very minimal as well, hence contributing very little to the differences in genome size. In addition, sequence variation analyses showed that *Bougainvillea* cp genomes share nearly identical genomic profiles though several potential barcodes, such as *ycf*1, *ndh*F, and *rpo*A were identified. Higher variation was observed in both *B. peruviana* and *B. pachyphylla* cp sequences based on SNPs and indels analysis. Phylogenetic reconstructions further showed that these two species appear to be the basal taxa of *Bougainvillea*. The rarely cultivated and wild species of *Bougainvillea* (*B. pachyphylla, B. peruviana, B. praecox*) diverged earlier than the commonly cultivated species and cultivar (*B. spectabilis, B. glabra, B.* cv.). Overall, the results of this study provide additional genetic resources that can aid in further phylogenetic and evolutionary studies in *Bougainvillea*. Moreover, genetic information from this study is potentially useful in identifying *Bougainvillea* species and cultivars, which is essential for both taxonomic and plant breeding studies.

## 1. Introduction

The family Nyctaginaceae, distributed primarily in the tropics and subtropics, contains around 400 species of trees, shrubs, and herbs classified in ca. 31 genera [1,2]. Nyctaginaceae has been well-recognized as one of the core groups of Caryophyllales (Centrospermae) based on the presence of betalain pigments, free-central placentation, p-type sieve tube elements, perisperm, and molecular evidence [1,3]. One of the most popular genera in Nyctaginaceae is *Bougainvillea*, a tropical and subtropical shrubby vine cultivated primarily for its colorful showy bracts. Their vibrant structures often mistaken as “flowers” are actually bracts or specialized leaves (ca. 0.5–2-inch long), in which the true flowers are attached at the mid-rib [4]. The true perfect flowers are normally small, tubular, white or yellowish in color, and surrounded by colorful petaloid bracts [4]. Due to *Bougainvillea*’s growth habit and attractive bracts, it became a widely known plant for landscaping [4]. It is commonly used in gardens as hedges or barriers, topiaries, and as ground cover on banks.

The horticulturally important species, such as *Bougainvillea glabra* and *Bougainvillea spectabilis*, are native to South America, but were brought and introduced to various countries all over the world. There are approximately 18 species of *Bougainvillea*, but only the above two species are well-known for cultivation [1,5]. The purpose of planting *Bougainvillea* is mostly ornamental, but recently a number of studies have explored the potential use of *Bougainvillea* as a medicinal plant and as a pollution-mitigating plant in industrial areas [6,7,8,9,10,11]. Recent research studies have tapped into the potential of *Bougainvillea* as an anti-inflammatory, anticancer, antioxidant, antimicrobial, and antihyperglycemic plant [7,8,9,10,11]. Specifically, *Bougainvillea spectabilis* has been well-known for its ability to lower blood sugar and improve liver function, while *Bougainvillea* cv. *buttiana* and *Bougainvillea glabra* exhibit significant anti-inflammatory activities [7,9,10]. The plant group has garnered research attention from the horticultural and pharmaceutical industries, and even in environmental studies. However, no recent studies have focused on the taxonomy of *Bougainvillea*, particularly regarding the wild species not utilized for cultivation.

Most of the publications on *Bougainvillea* focused on taxonomic descriptions were published decades ago [12,13,14], and no actual revision of the topic has been attempted since then. Molecular studies based on short-fragment sequences centered on Nyctaginaceae include only a few sequences of *Bougainvillea*. Phylogenetic studies on family Nyctaginaceae based on *ndh*F, *rps*16, *rpl*16, and nrITS revealed that *Bougainvillea glabra* and *Bougainvillea infesta* are actually closely related to *Belemia* and *Phaeoptilum* [15]. Similarly, the phylogenomic study about the order Caryophyllales includes only *Bougainvillea spectabilis* as representative of Bougainvilleeae [16]. To date, no phylogenetic study has been published focusing specifically on *Bougainvillea* species.

Even though next generation sequencing (NGS) has instigated a rapid increase in the available complete organelle genome sequences of plants that can be used for phylogenetic studies, sequences of *Bougainvillea* are rarely deposited in the database. The commercial value of *Bougainvillea* as an ornamental plant has overshadowed the need for genetic studies. Hence, the majority of the available sequences in the GenBank are from the commercially cultivated *Bougainvillea*. Short-genome announcement papers have presented the gene content and structure of *B. glabra* and *B. spectabilis* [17,18]. It was also shown that *Bougainvillea* is clustered together with *Acleisanthes, Mirabilis,* and *Nyctaginia*; however, specific relationships cannot be inferred due to the lack of available sequences [17]. Currently, there is very little information available on the genetic structures of *Bougainvillea*, particularly on their plastome features and specific phylogenetic placements.

It has been established that plastomes, particularly chloroplast genomes, are useful in phylogenetic studies due to their ability to self-replicate, their conservative structure, and their slow evolutionary rate [19]. Investigating the genome organization and genetic information for plastid genomes provide scientists relevant data that can be utilized for species conservation, phylogenetic reconstruction, molecular marker development, genomic evolution studies, and for solving taxonomic complexities in different taxa [19,20,21]. Therefore, sequencing additional cp genomes of *Bougainvillea* species is an initial step that is needed to fill the gap in genomic resources before conducting further studies. Thus, this study aimed to characterize the cp genome sequences of several cultivated and wild *Bougainvillea* species (*Bougainvillea glabra, Bougainvillea peruviana, Bougainvillea pachyphylla, Bougainvillea praecox*, and *Bougainvillea* cv.). Specifically, the study aimed to discuss the structures and features of the five newly sequenced cp genomes, including the codon usage, RNA editing, simple sequence repeats, tandem repeats, IR contractions and expansion, divergent regions, SNPs, and indels. Phylogenetic analyses were also conducted to determine the relationship among cultivated and wild *Bougainvillea* in the family Nyctaginaceae. 

## 2. Results and Discussion

### 2.1. General Features of Bougainvillea Chloroplast Genomes

**Figure 1:**
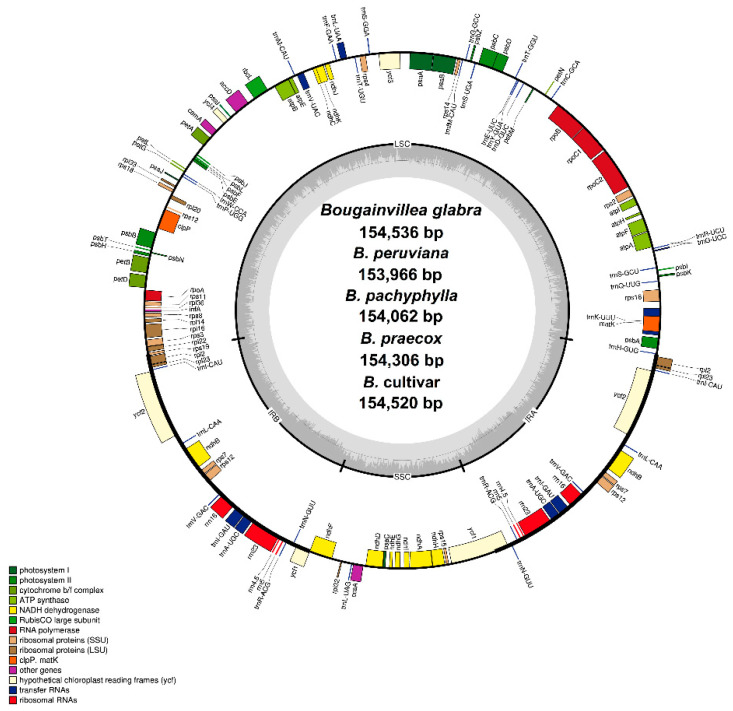
Circular gene map of five newly sequenced *Bougainvillea* chloroplast genomes. The genes drawn outside the circle are transcribed clockwise, while the genes on the inside are transcribed counterclockwise. Genes belonging to different functional groups are color-coded. The dark gray plot in the inner circle represents the GC (Guanine-Cytosine) content, whereas the light-grey corresponds to the AT (Adenine-Thymine) content.

The assembled chloroplast genome sequences of *Bougainvillea glabra*, *Bougainvillea peruviana*, *Bougainvillea pachyphylla*, *Bougainvillea praecox*, and *Bougainvillea* cv., together with the available sequence of *Bougainvillea spectabilis*, range from 153,966 bp to 154,541 bp. Similar to most sequenced Nyctaginaceae cp genomes, they all exhibit the typical quadripartite structure, consisting of a large single-copy (LSC) region (85,159–85,708 bp), a small single-copy (SSC) region (18,014–18,078 bp), and a pair of inverted repeats (25,377–25,427 bp) (Figure 1, Table 1). The commonly cultivated *Bougainvillea spectabilis* and *Bougainvillea glabra* have the largest cp genomes, but despite the differences in size, all cp genomes have a total of 131 genes, including 86 protein-coding genes, eight rRNA, and 37 tRNA. Of the identified genes, there are seven protein-coding (*ndh*B, *rpl*2, *rpl*23, *rps*7, *rps*12, *ycf*1, *ycf*2), four rRNA (*rrn*4.5, *rrn*5, *rrn*16, *rrn*23), and seven tRNA (*trn*I-CAU, *trn*L-CAA, *trn*V-GAC, *trn*I-GAU, *trn*A-UGC, *trn*R-ACG, *trn*N-GUU) genes duplicated in the IR regions. Thus, there are 79 unique protein-coding genes that function primarily in photosynthesis and transcription–translation processes, while the remaining are transfer RNA genes (30), and ribosomal RNA genes (four) (Table 2). The numbers of rRNA and tRNA genes are highly conserved among Nyctaginaceae, but the number of protein-coding genes differs due to acetyl-CoA carboxylase subunit D gene (*acc*D) loss in some genera. *Bougainvillea* cp genomes have an intact *acc*D gene, whereas *Nycataginia capitata* (MH286318.1) and *Pisonia aculeata* (MK397866.1) lack the *acc*D gene. In general, the genome structure and features of the *Bougainvillea* cp genomes are highly similar to most Nyctaginaceae chloroplast genomes—no significant changes in the gene order or gene content have been observed.

In addition, there are also 17 intron-containing genes identified in all six cp genomes, of which 15 genes (*rps*16, *atp*F, *rpo*C1, *pet*B, *pet*D, *rpl*16, *rpl*2, *ndh*B, *ndh*A, *trn*I-GAU, *trn*A-UGC, *trn*V-UAC, *trn*L-UAA, *trn*G-UCC, *trn*K-UUU) have one intron, while *clp*P and *ycf*3 have two introns each (Table 2). The *trn*K-UUU gene has the longest intron (2508–2524 bp), which encodes the *mat*K ORF. This is normally perceived in published cp genomes, since plastid *trn* introns are relatively longer in comparison to nuclear tRNA introns [22]. A trans-splicing event also occurs in the *rps*12 gene, with the 5′ exon positioned in the LSC region, while the 3′ exons are duplicated in the IR regions. The aforementioned genes with introns can be categorized into three types—genes for electron transfer, protein synthesis, and ATP synthesis. Most introns in general do not code for proteins, but recent studies have shown that they can enhance gene expression and regulation in specific locations [23,24]; hence, they are potential sites for efficient processing of native or foreign transcripts that can improve particular horticultural traits in plants such as *Bougainvillea*.

Overall, the GC contents of the six *Bougainvillea* cp genomes are almost identical, ranging from 36.4% to 36.6%. Interestingly, the GC contents of the IR regions (42.7–42.8%) are higher compared to the LSC (34.2–34.3%) and SSC regions (29.5–29.6%). Higher GC contents in IR regions are typically linked to the presence of rRNA genes in the IRs or to the GC-biased conversion (gBGC) [20]. The gBGC is a preferential fixation of AT to GC mutations over GC to AT mutations, thus increasing the GC contents in recombination hotspots such as IR regions [25,26,27].

### 2.2. Codon Usage Analysis

**Figure 2:**
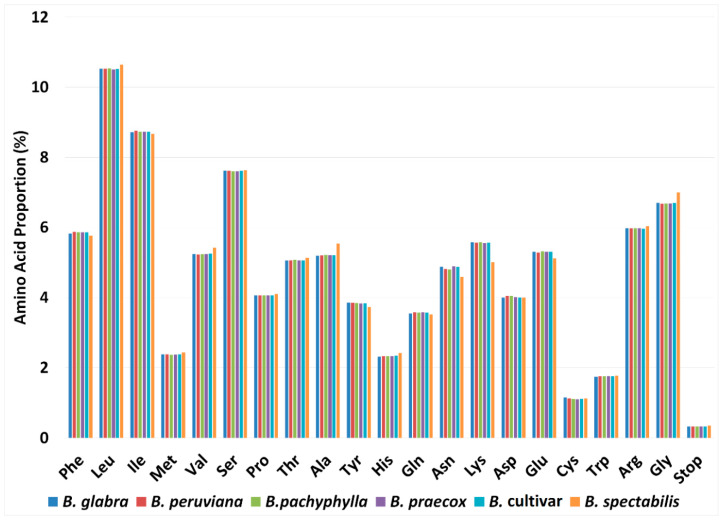
Percentages of amino acid in the protein-coding regions of six *Bougainvillea* chloroplast genomes.

Approximately 48–52% of the six *Bougainvillea* cp genomes are comprised of protein-coding genes with 24,557–26,717 codons. Of these codons, leucine (10.50–10.64%) and isoleucine (8.67–8.76%) are the most abundant amino acids, whereas cysteine (1.10–1.15%) has the lowest frequency (Figure 2). The high leucine frequency can be attributed to the fact that leucine biosynthesis is greatly needed in chloroplasts, due to its important function in photosynthesis-related metabolism [28]. On the other hand, cysteine is quite reactive and considered toxic if it is allowed to accumulate above a certain level [29]. It is also highly susceptible to changes in biological conditions [30]. This pattern is highly uniform in most angiosperm cp genomes [21,31].

In addition, codon usage analysis also revealed that there are particular amino acid codons that are more frequently used or preferred [32]. This codon usage bias has been commonly observed in plant genomes, and it is assumed that preferred codons are normally utilized in highly expressed genes [33]. Based on the relative synonymous codon usage values (RSCU), all amino acid codons found in the six *Bougainvillea* cp genomes exhibit codon preferences, except for tryptophan (UGG) and methionine (AUG) (RSCU = 1). There are 30 codons that are highly favored (RSCU > 1) and 32 codons that are less preferred (RSCU < 1). Moreover, it can be observed that out of the 30 preferred codons, 29 are A/U-ending codons, meaning that C/G-ending codons are less common in the chloroplast genomes. Several reported cp genomes from other families such as Zingiberaceae, Euphorbiaceae, and Asparagaceae have constantly reported the same occurrence [31,34,35]. The bias towards high A/U occurrences in the third nucleotide position of codons appears to be conserved among higher plants.

### 2.3. RNA Editing Sites

Nucleotide sequences in cpDNAs are commonly altered at the transcript level through RNA editing and RNA splicing. Thus, determining the RNA editing sites is necessary in order to understand coding information in the cp genomes. Putative RNA editing sites in the six *Bougainvillea* cp genomes were predicted using the Predictive RNA Editor for Plants (PREP) software, which identified a total of 42 RNA editing sites. Parallel to other seed plants, the annotated RNA editing sites in the *Bougainvillea* cp genomes were C to U conversions located in the first or second position of the codons (Table S2). C to U editing mainly occurred in chloroplast and mitochondrial genomes of angiosperms and gymnosperms, although U to C conversions were also observed in ferns and bryophytes [36]. Consistent with prior studies, most of the editing sites were observed to be distributed mainly in the *ndh* genes (*ndh*A, *ndh*B, *ndh*D, *ndh*F, *ndh*G) of the cp genomes, particularly in *ndh*B (12 sites) [36,37]. In addition, most of the RNA editing amino acids have a tendency to be converted from serine (S) to Leucine (L), and the majority of the changes were from hydrophilic to hydrophobic. These results indicate that RNA editing increases hydrophobicity, which might influence the proteins’ secondary or tertiary structures [36,38]. RNA editing can actually lead to re-establishment of conserved amino acid residues, increases in hydrophobicity, and regulation of protein expression [36,38].

### 2.4. Simple Sequence Repeats and Tandem Repeat Analyses

**Figure 3:**
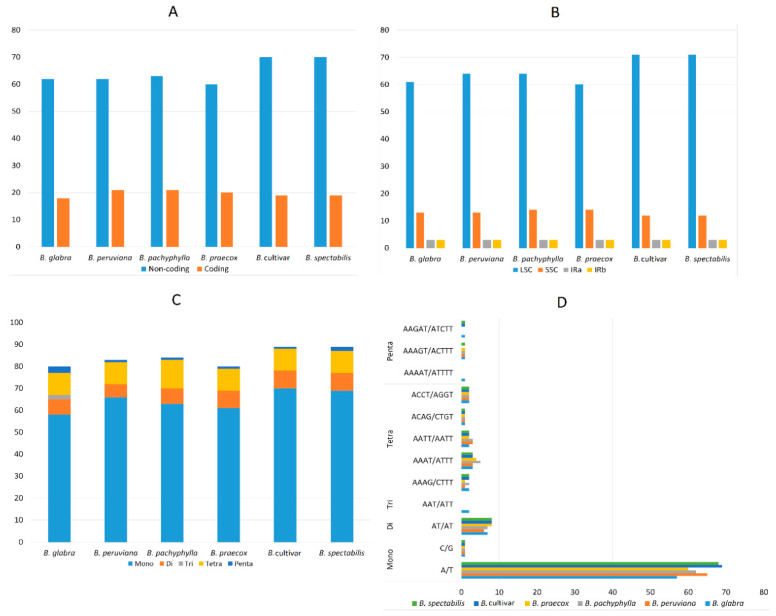
Analysis of simple sequence repeats (SSRs) in six *Bougainvillea* chloroplast genomes. (**A**) Simple sequence repeats detected in coding and non-coding regions of six *Bougainvillea* cp genomes. (**B**) Simple sequence repeats distributions in the LSC, SSC, and IR regions of *Bougainvillea* cp genomes. (**C**) Numbers of different types of SSRs identified in the *Bougainvillea* cp genomes. (**D**) Frequency of various SSR types identified in six *Bougainvillea* cp genomes.

The analysis of repetitive sequences identified a total of 80, 83, 84, 80, 89, and 89 chloroplast simple sequence repeats (cpSSRs) in *Bougainvillea glabra, Bougainvillea peruviana, Bougainvillea pachyphylla, Bougainvillea praecox, Bougainvillea* cv., and *Bougainvillea spectabilis*, respectively (Figure 3A–C). *Bougainvillea spectabilis* and *Bougainvillea* cv. have the highest numbers of cpSSRs, while *Bougainvillea glabra* and *Bougainvillea praecox* have the lowest numbers of cpSSRs. In agreement with other studies, about 71.25–78.3% of the identified cpSSRs are A/T mononucleotides, and most of the dinucleotides, trinucleotides, tetranucleotides, and pentanucleotides contain A/T, hence contributing to the AT richness of *Bougainvillea* cp genomes (Figure 3D). Trinucleotides are rare in *Bougainvillea* cp genomes—only *B. glabra* has trinucleotides detected in its cp genome. Chloroplast simple sequence repeats are also primarily found in the intergenic spacers of the LSC region (Figure 3A,B). The frequency of cpSSRs in coding regions is relatively lower, since the rate of mutation in cpSSRs is higher and might affect gene expression [39]. As mentioned in earlier studies, cpSSRs in the non-coding regions typically exhibit intraspecies differences in repeat number [40]. SSRs are also considered as a popular chloroplast marker due to their high level of polymorphism, co-dominance mode of inheritance, and multi-allelic nature [41]. Therefore, identified cpSSRs can be potentially useful for population studies or phylogeographic studies of *Bougainvillea* species and their cultivars. The initial cpSSR study conducted in fifty cultivars of *Bougainvillea* verified that SSRs can be used for molecular characterization and identification of cultivars [41].

**Figure 4:**
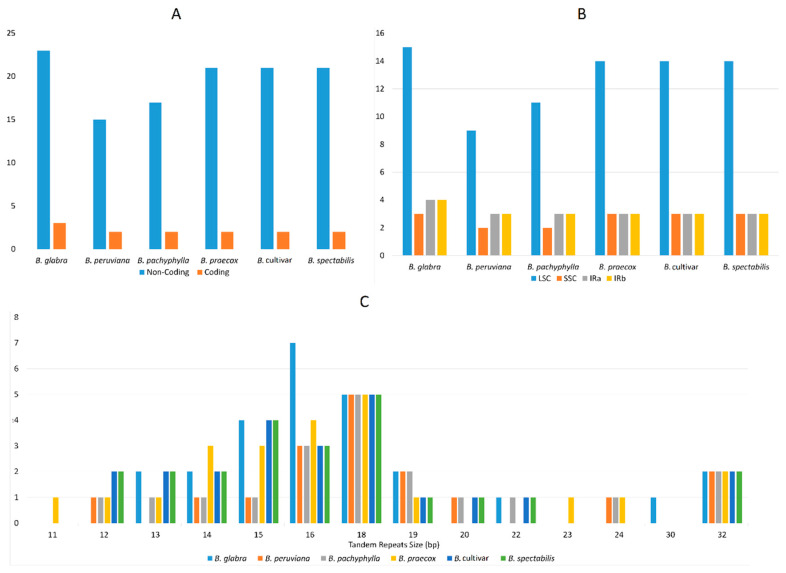
Tandem repeat analysis in six *Bougainvillea* cp genomes. (**A**) Frequency of tandem repeats in the non-coding and coding regions of six cp genomes. (**B**) Distributions of the detected tandem repeats in LSC, SSC, and IR regions. (**C**) Lengths of the identified tandem repeats in all six *Bougainvillea* cp genomes.

In addition to SSRs, tandem repeats (TRs) were also determined in the six cp genomes using Tandem Repeats Finder v4.04. *Bougainvillea glabra* (26) has the highest number of identified tandem repeats, whereas *Bougainvillea peruviana* (17) and *Bougainvillea pachyphylla* (19) have the lowest. The remaining three (*B. praecox, B. spectabilis, B. cultivar*) have the same number of tandem repeats. Similar to SSR, TRs in the six cp genomes are mostly distributed in the non-coding areas of the LSC region (Figure 4A,B). The lengths of the identified TRs range from 11 to 32 bp, but they are mainly around 15–18 bp (Figure 4C). All TRs found in the coding regions of six genomes are located in the hypothetical chloroplast reading frame *ycf*2. A higher density of TRs in non-coding regions is quite common in angiosperms, since mutations in TRs situated in the known protein-coding regions can result in protein function changes [42]. TR mutations occur due to modifications in the number of repeating units, which can possibly cause unfavorable phenotypes [43].

### 2.5. IR Contraction and Expansion

**Figure 5:**
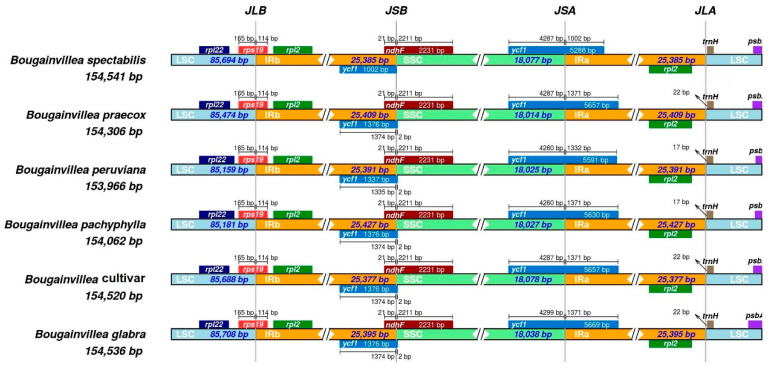
Comparisons of LSC, SSC, and IRs junctions among the six chloroplast genomes.

Inverted repeat regions are generally conserved among land plants. They all contain four rRNA, five tRNA genes, and a few protein-coding genes as a result of some expansion and contraction in the IR junctions [44]. Sequences flanking the IR junctions may vary among different species, which might result in genome size variation. In *Bougainvillea* cp genomes, the gene contents and arrangements are highly similar, however there are few contractions and expansions in the IR boundaries (Figure 5). For instance, the IRb–LSC junction (JLB) is situated within the *rps*19 gene in all *Bougainvillea* cp genomes, thus *rps*19 has a 114 bp extension in the IRb region. In addition, all taxa have the *ycf*1 gene in the IRa–SSC junction (JSA), hence producing long fragments of *ycf*1 in the IRb–SSC junction (JSB). It is also evident that the partial copies of *ycf*1 in the IRb regions of all cp genomes overlap with the *ndh*F gene. However, in five *Bougainvillea* cp genomes (*B. glabra, B. peruviana, B. pachyphylla, B. praecox, B.* cv.), the *ycf*1 fragments have two bp extension in the IRb–SSC junction (JSB), suggesting an infinitesimal expansion of the IR. This IR contraction and expansion pattern is usually observed in most angiosperms [31,44,45]. All of the sequences used in this study belong to the same genus, so the IR boundary shifts are relatively minor, hence contributing very little to the observed differences in genome size.

### 2.6. Sequence Variation Analyses among Bougainvillea cp Genomes

**Figure 6:**
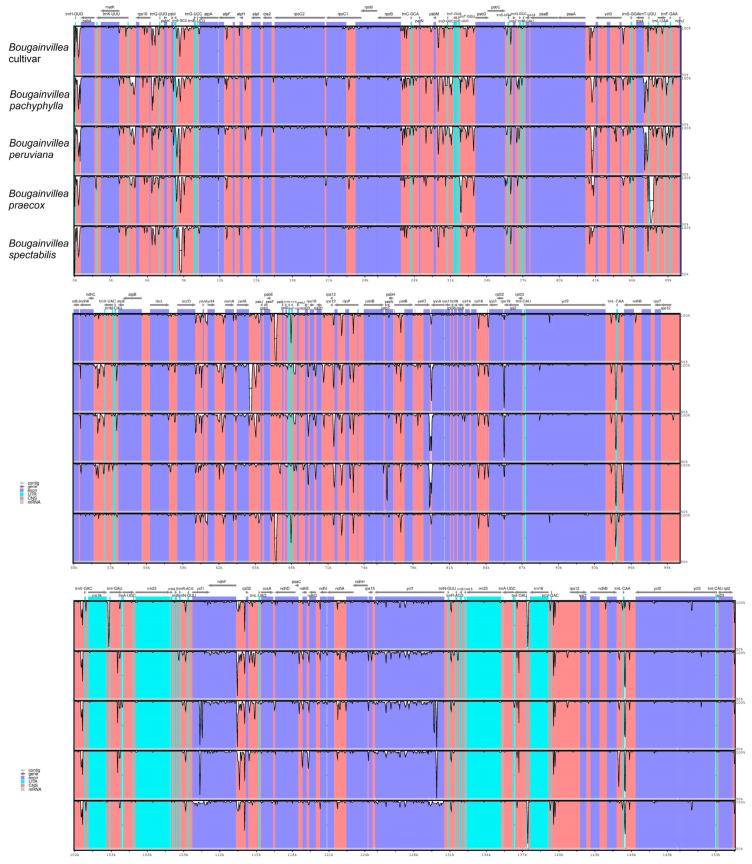
Sequence identity plots (mVISTA) among *Bougainvillea* species. Alignments of the five *Bougainvillea* plastomes, with *Bougainvillea glabra* as the reference genome. Genes are color-coded, whereby pink regions represent conserved non-coding sequences (CNS) and purple regions indicate protein-coding sequences. Grey arrows above the alignments indicate gene directions. The y-axis denotes the percentages of identity, ranging between 50% and 100%.

Sequence divergence among *Bougainvillea* cp genomes was compared through multiple sequence alignment carried out in mVISTA. Generally, no significant rearrangements were observed among *Bougainvillea* cp genomes, but several regions displayed higher variation than others. Resulting alignment analysis using *Bougainvillea glabra* as a reference showed that the coding regions of the *Bougainvillea* cp genomes are less divergent compared to the non-coding regions, whereas the non-coding regions are more variable than the coding regions (Figure 6). Likewise, the IR regions have lower divergence and more conserved compared to LSC and SSC regions. Specifically, protein-coding genes such as *ycf*1 and *ndh*F and non-coding regions such as start-*psb*A, *rps*16-*psb*K, *psb*I-*atp*A, *psa*A-*ycf*3, *pe*tD-*rpo*A, and *ndh*F-*rpl*32 are considered to be highly divergent regions.

**Figure 7:**
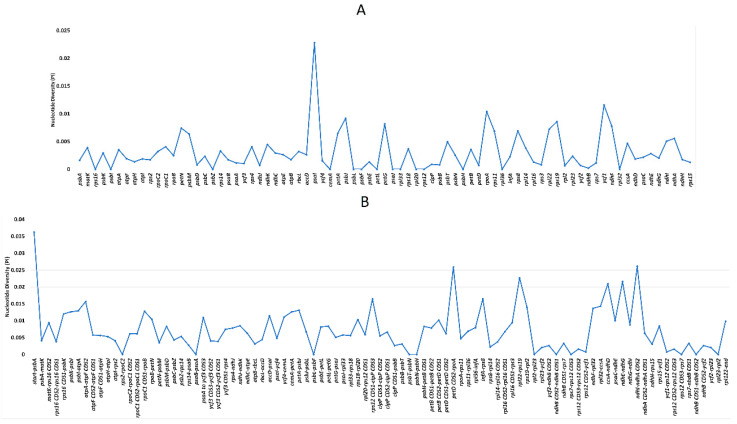
Nucleotide diversity (Pi) of various regions in *Bougainvillea* chloroplast genomes. (**A**) Nucleotide diversity values in the protein-coding regions. (**B**) Nucleotide diversity values in the non-coding regions.

To further examine the sequence divergence in the *Bougainvillea* cp genomes, nucleotide diversity (Pi) values were calculated using DnaSP v5.10 (Figure 7). Similar standards to those employed in the Zingiberaceae family were used to determine the divergence hotspots in coding and non-coding regions [31]. For the 79 unique protein-coding genes, the nucleotide diversity values range from 0 to 0.2282, with an average of 0.00307. Eleven protein-coding genes (*pet*N, *psa*I, *psb*J, *pet*G, *rpo*A, *rps*8, *rps*11, *rpl*22, *rps*19, *ycf*1, *ndh*F) positioned at the single copy regions exhibit higher Pi values (>0.005) (Figure 7A). On the other hand, the Pi values of the non-coding regions range from 0 to 0.03629, with an average of 0.00798. Among these regions, 10 regions (start-*psb*A, *rps*16 CDS1-*psb*K, *psb*K-*psb*I, *psbI*-*atp*A, *cem*A-*pet*A, *pet*D CDS2-*rpo*A, *ndh*F-*rpl*32, *rpl*32-*ccs*A, *ndh*E-*ndh*G, *ndh*I-*ndh*A CDS2) have high diversity values (>0.012) (Figure 7B). Most of these divergence hotspots are located in the LSC and SSC regions, signifying that IR regions are less variable. These results also exemplify that most hypervariable regions shown in the mVISTA alignment have higher nucleotide diversity (Pi) values as well.

**Figure 8:**
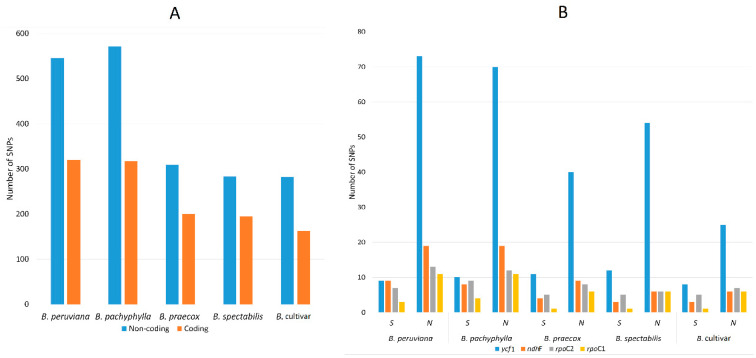
Summary of SNPs detected in the five *Bougainvillea* chloroplast genomes. (**A**) Frequency of SNPs in the coding and non-coding regions. (**B**) Protein-coding genes with highest numbers of synonymous and non-synonymous SNPs.

The overall variation among the *Bougainvillea* sequences was analyzed through mVISTA and DnaSP v5.10, however to further elucidate the differences among taxa, SNP/indel analysis was also conducted using MUMmer 4 and Geneious 2020.2. Using *Bougainvillea glabra* as a reference, the single-nucleotide polymorphisms (SNPs) and indels (insertions deletions) were identified in the other five *Bougainvillea* cp genomes. General results revealed that more SNPs and indels were detected in *B. pachyphylla* and *B. peruviana* in contrast to the other three plastomes. When aligned to *B. glabra*, *B. pachyphylla* has 571 SNPs in the non-coding regions and 317 SNPs in the coding regions, whilst *B. peruviana* has 545 SNPs in the non-coding regions and 320 SNPs coding regions (Figure 8A). Lesser SNPs were found in *B. praecox* (309, 200), *B. spectabilis* (283, 195), and *B*. cultivar (282, 163), indicating that these three have higher sequence similarities to *B. glabra* (Figure 8A). There are also around 45–58 protein-coding regions with SNPs; specifically, *ycf*1, *ndh*F, *rpo*C2, and *rpo*C1 have higher numbers of SNPs (Figure 8B, Table S3). In all *Bougainvillea*, the *ycf*1 gene has the highest numbers of both synonymous and non-synonymous SNPs. The RNA polymerase genes (*rpo*A, *rpo*B, *rpo*C2, *rpo*C1), particularly *rpo*C1 and *rpo*C2, predominantly contain synonymous and non-synonymous SNPs (Table S3). Similar to the mVISTA alignment and nuclear diversity analyses, *ycf*1 and *ndh*F are also considered highly variable due to their high numbers of SNPs. 

**Figure 9:**
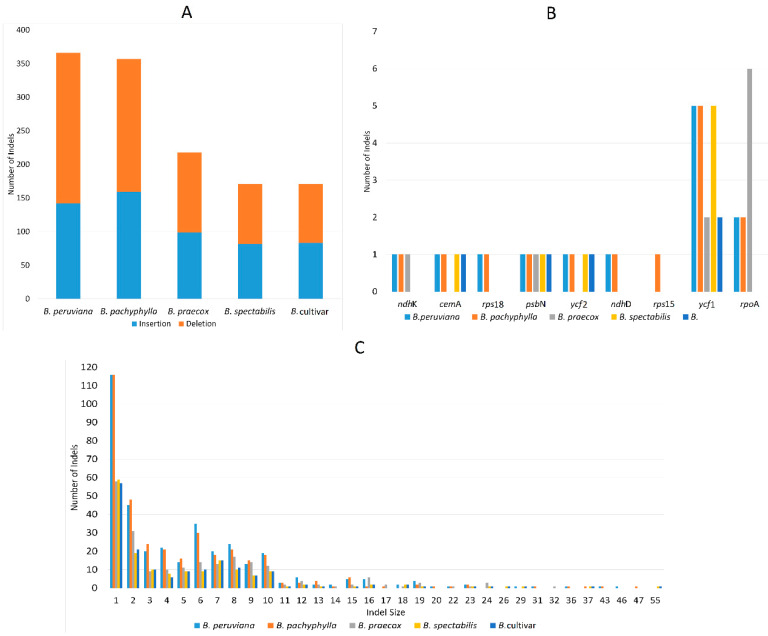
Summary of insertions and deletions found in five *Bougainvillea* cp genomes. (**A**) Total number of indels in five *Bougainvillea* species. (**B**) Numbers of indels located in the protein-coding genes. (**C**) Lengths of indels identified in five cp genomes.

A similar trend can be observed in *Bougainvillea* indels, *B. peruviana* (366), and *B. pachyphylla* (357), which have more indels in comparison to *B. praecox* (218), *B. spectabilis* (171), and *B*. cultivar (171) (Figure 9A). Both *B. spectabilis* and *B*. *cultivar* have lesser indels, indicating less differences from *B. glabra*. The presence of the large deletion in the *clp*P intron of *B. spectabilis* (55 bp), *B. cultivar* (55 bp), *B. pachyphylla* (47 bp), and *B. peruviana* (29 bp) mainly differentiates these four from *B. glabra* (Figure S1). On the other hand, both *B. peruviana* and *B. pachyphylla* differ from *B. glabra* by having a 43 bp deletion in the spacer in between *rpl*22 and *rps*19 (Figure S2). Although small indels are quite common in all *Bougainvillea* cp genomes, more one-bp indels (116) are discovered in *B. peruviana* and *B. pachyphylla* (Figure 9C). The presence of copious amounts of small indels and several large indels results in high sequence variation in *B. peruviana* and *B. pachyphylla* (Figure 9A,C). In addition, the majority of the indels detected in the five cp genomes are in the non-coding regions—only *ycf*1, *ycf*2, *cem*A, *ndh*K, *ndh*D, *rpo*A, *psb*N, and *rps*15 have indels (Figure 9B). Again, *ycf*1 and one of the RNA polymerase genes (*rpo*A) exhibit a high degree of variation due to the presence of indels.

Consolidating the results from various analyses, regions such as *ycf*1, *ndh*F, and *rpo*A are potential molecular markers. Consistent with all analyses, *ycf*1 has high sequence divergence, high diversity value, and has the most SNPs and indels (Figure 6, Figure 7A, Figure 8B, and Figure 9B). Another good barcode candidate is the *ndh*F gene, which displays high sequence divergence, high Pi value, and high SNP density (Figure 6, Figure 7A, and Figure 8B). Likewise, the nucleotide diversity analysis also showed that *rpo*A is considered a variable region (Figure 7A). Additionally, it also has good amounts of detected SNPs and indels (Table S2, Figure 9B). Therefore, to check whether these regions can actually differentiate *Bougainvillea* species, phylogenetic analyses was also conducted and compared to the phylogenetic tree inferred from all the protein-coding regions in *Bougainvillea* cp genomes.

### 2.7. Phylogenetic Analysis

**Figure 10:**
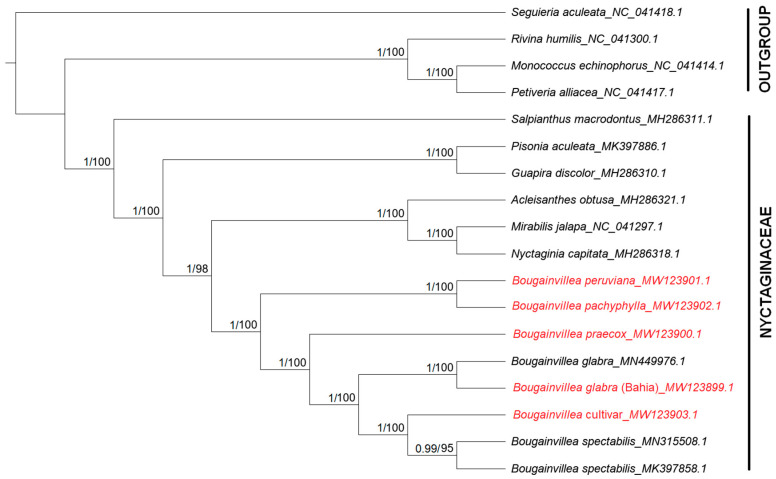
Maximum Likelihood (ML) and Bayesian Inference (BI) consensus tree based on the 79 concatenated protein-coding regions of 14 Nyctaginaceae cp genomes. Species from Petiveriaceae were used as outgroups. Numbers on each node represent bootstrap support and Bayesian posterior probability (BPP) values. Branches with bootstrap values > 75 and BPP values > 95 are considered as highly supported.

Several of the phylogenetic reconstructions focusing on the Nyctaginaceae family were based on a single gene or a few gene regions from plastid or nuclear DNA [15,46], but none of them explicitly discussed the relationships among species of *Bougainvillea*. With the advent of next-generation sequencing, several cultivated *Bougainvillea spectabilis* and *Bougainvillea glabra* genomes have been published, but due to the lack of other available sequences, phylogenetic analyses have focused only on *Bougainvillea’s* placement within the Nyctaginaceae family [17,18]. Thus, in this study, the phylogeny of *Bougainvillea* was reconstructed using eight complete chloroplast genomes of *Bougainvillea* (including the sequences from GenBank) and six other species from the Nyctaginaceae family. Species from Petiveriaceae were used as outgroups. In addition, the highly variable regions from the same dataset were extracted and used to construct a phylogenetic tree. As a whole, the resulting ML and BI trees based on complete cp genomes have a consistent and well-supported topology (Figure 10). Likewise, a similar topology was observed for the ML tree generated from the potential markers obtained from the sequence variation analyses (Figure S3). 

Bougainvilleeae, as represented by the *Bougainvillea* species, has higher affinity to the Nyctagineae tribe (*Acleisanthes, Mirabilis, Nyctaginia*) compared to other tribes within Nyctaginaceae. Within the *Bougainvillea* genus, *Bougainvillea peruviana* and *Bougainvillea pachyphylla* appear to be the basal taxa, while *Bougainvillea praecox* is a sister to two distinct subclades: the “glabra” and “spectabilis” subclades. 

Based on its morphology, *B. peruviana* was assumed to be closely related to either *B. glabra* [12,47] or *B. pachyphylla* [48], but molecular data from cp genomes revealed that it has a closer relationship to *B. pachyphylla*. The sequence variation analysis discussed earlier also showed that *B. peruviana* and *B. pachyphylla* are the two genomes that differ most from *B. glabra*. In terms of morphological structure, *B. pachyphylla* is not too distinct from *B. peruviana*—it differs only by having thick and leathery leaves and a densely puberulent perianth in comparison to *B. peruviana* [48,49]. *B. peruviana* is considered one of the most stable species, as there is less variation in the shapes of the bracts and leaves [4]. This species is also not as vigorous as the cultivars and hybrids [4]. Therefore, the striking morphological and molecular similarities of *B. pachyphylla* to *B. peruviana* must be taken into consideration in future taxonomic revisions for the genus *Bougainvillea*. 

On the other hand, *Bougainvillea praecox* is actually distinct from other *Bougainvillea* by being sparsely spiny or unarmed [12]. It is not usually used for cultivation, but it has showy white bracts that become greenish when dried. Based on the tree, *B. praecox* is the sister to two distinct groups containing *Bougainvillea glabra* and *Bougainvillea spectabilis*.

The close relationship between *Bougainvillea glabra* and *Bougainvillea* spectabilis is not surprising, as morphologically *B. glabra* is highly similar to *B. spectabilis* [10]. *B. glabra* differs only by having puberulent to glabrate branches and leaves, while *B. spectabilis* has fulvous–villous branches and densely villous abaxial leaf surfaces [12,49]. In addition, horticulturists have observed that *B. spectabilis* has stouter spines and wavy bracts. The sequences from GenBank are mostly cultivated and are either classified as *B. glabra* or *B. spectabilis*. Similarly, the *Bougainvillea* cultivar clusters together with *B. spectabilis* and *B. glabra*, since most of the cultivars are actually crosses between *B. spectabilis* and *B. glabra*. Many crosses between the two species have produced new hybrids and horticultural cultivars. As presented earlier, less sequence variation was observed among these three species, so closer relationships are also evident in the resulting trees.

In general, it can be observed that rarely cultivated and wild species of *Bougainvillea* (*B. pachyphylla, B. peruviana, B. praecox*) diverged earlier than the commonly cultivated species of *Bougainvillea*. These results showed that information inferred from highly variable regions and complete cp genome sequences resulted in consistent phylogeny. The potential barcodes can also successfully differentiate *Bougainvillea* species and cultivars, meaning more samples can be sequenced to give a broader view of the evolutionary relationships within this genus.

## 3. Materials and Methods

### 3.1. Plant Samples and DNA Extraction

The leaf samples of the four *Bougainvillea* species and one cultivar used in this study were collected from Brazil, Peru, Ecuador, and China. Two samples were obtained from Brazil, namely *Bougainvillea glabra* (M.B.M. da Cruz 0001 NY) from Ilhéus, Bahia; and *Bougainvillea praecox* (Chen T. 2012063001 SZG) from Jardim Botanico Plantarum, Nova Odes. *Bougainvillea pachyphylla* (Sagastegui A. et al. 15924 MO) was from Chota, Cajamarca, Peru, while *Bougainvillea peruviana* (Chen T. et al. 2014052606 SZG) was collected on the way from Loja to Macara, Ecuador. Lastly, the *Bougainvillea* cultivar (Chen T. 2020031204 SZG), which is originally from India, was acquired from Fairy Lake Botanical Garden, Shenzhen, China. Fresh leaves of *B. glabra*, *B. praecox*, *B. peruviana*, and *B. cultivar* were dried in silica gel, while the *B. pachyphylla* leaf sample was obtained from herbarium material. Total genomic DNA was extracted from each sample through the modified CTAB (cetyl trimethylammonium bromide) method [50], then the DNA quality was checked through agarose gel electrophoresis, nanodrop method, and Qubit 2.0.

### 3.2. Chloroplast Genome Sequencing, Assembly, and Annotation

After the DNA quality assessment, DNA was sheared to fragments using a Covaris ultrasonic disruptor. Short-insert (350–400 bp) libraries were constructed using the Nextera XT DNA Library Preparation Kit. Sequencing was performed in Illumina Novseq 600 platform, and for each sample around 10.0 Gb of raw data were generated, with an average read length of 150 bp and sequencing depths of 263.8X–1627.3X. The Illumina raw sequence reads were filtered for adaptor sequences, undersized inserts, duplicated reads, and low-quality reads using the NGS-QC (Next Generation Sequencing Quality Control) toolkit [51]. High-quality reads were assembled into contigs via the de novo assembler SPAdes 3.11.0, using a k-mer set of 93, 105, 117, 121 [52].

The assembled *Bougainvillea* cp genomes were annotated using Plann software [53] and the online annotation tools cpGAVAS [54] and DOGMA [55]. Protein-coding gene annotation was also verified by BlastN searches of the non-redundant database at the National Center for Biotechnology Information (NCBI). RNAmmer 1.2 [56] and tRNAscan-SE v2.0 [57] were used to annotate rRNA and tRNA genes, respectively. The genome maps of the *Bougainvillea* cp genomes were generated using the online program OGDRAW v1.3.1 [58], then the cp genome sequences were deposited in National Center for Biotechnology Information (NCBI) GenBank with the accession numbers MW123899–MW123903.

### 3.3. Codon Usage and RNA Editing Sites Prediction

Relative synonymous codon usage (RSCU) was determined for all protein-coding genes using MEGAX software [59]. Amino acid frequency values were also obtained from MEGA X [59] and manually verified. In addition, probable RNA editing sites in six *Bougainvillea* cp genomes were identified using Predictive RNA Editor for Plants (PREP) suite (http://prep.unl.edu/), with a cutoff value of 0.8 [60]. The default settings (35 coding sequences) were used to predict putative RNA editing sites.

### 3.4. Repeat Analysis

Simple sequence repeats (SSRs) in the *Bougainvillea* cp genomes were identified using MISA (http://pgrc.ipk-gatersleben.de/misa/) [61], with motif sizes of one to six nucleotides and thresholds of 10, 5, 5, 3, 3, and 3. Similar parameters had been used in other angiosperm cp genomes. Aside from SSRs, tandem repeats were also analyzed with the aid of Tandem Repeats Finder Program v4.04 [62] using default parameters. All identified repeats were manually filtered and redundant results were excluded. For tandem repeats, repeats with more than 90% sequence identity were included. 

### 3.5. Genome Comparison and Divergence Analyses

The five newly sequenced *Bougainvillea* cp genomes were compared to the available cpDNA sequence of *Bougainvillea spectabilis*. For comparison, sequence alignment was carried out in mVISTA (http://genome.lbl.gov/vista/mvista/about.shtml) using Shuffle-LAGAN mode [63]. *Bougainvillea glabra* was used as the reference. In addition, the nucleotide diversity (Pi) values of the cp genomes were determined to detect various divergence hotspots. Using MAFFT v7.388 [64], *Bougainvillea* cp sequences were aligned, then DnaSP v5.10 [65] was utilized to compute the nucleotide diversity (Pi) values. Sliding window analysis was used, with a window length of 600 bp and step size of 200 bp. The expansions and contractions in the IR junctions were also depicted using the online tool IRscope (https://irscope.shinyapps.io/irapp/) [66]. To further examine variations among the *Bougainvillea* cp genomes, SNPs and indels were also identified and located using MUMmmer 4 [67] and Geneious Prime 2020.2 [68]. The *Bougainvillea glabra* cp genome was also used as the reference.

### 3.6. Phylogenetic Analyses

Together with the five newly sequenced *Bougainvillea* cp genomes, three available *Bougainvillea* sequences and six Nyctaginaceae sequences deposited in NCBI GenBank were included in the dataset. Four complete cp genomes from the closely related family Petiveriaceae were used as outgroups. From these sequences, 79 protein-coding regions were extracted using Geneious Prime 2020.2. Multiple alignments of these extracted regions were conducted in MAFFT v7.388 [64]. Ambiguous regions in the alignments were manually removed, then the filtered alignments were concatenated using Geneious Prime 2020.2 [68], which generated a final alignment of 66,426 bp. Aside from the 79 concatenated protein-coding regions, sequenced alignment was also conducted in the selected highly divergent regions to test their barcode effectivity. The genes *ndh*F, *rpo*A, and *ycf*1 were extracted and concatenated using Geneious Prime 2020.2 [68]

Maximum likelihood (ML) analyses were performed using RAxML 8.2.11 [69] and IQ-TREE v6.10 [70] with the GTR+I+G (General Time Reversible + Invariable Sites + Gamma Distribution) nucleotide substitution model. The best fit model was determined through jModelTest2, executed in CIPRES Gateway [71]. The bootstrap consensus tree was inferred from 1000 replicates. Furthermore, Bayesian inference tests were also conducted in MrBayes 3.2.6 [72] with the general time reversible (GTR) DNA substitution model and the gamma distribution rate variation across sites. Bayesian Inference analyses were carried out in CIPRES Gateway [71], with four Markov chain Monte Carlo (MCMC) running for one million generations, with sampling every 1000 generations and the first 25% being discarded as burn-in. The resulting branches with bootstrap support > 75% for maximum likelihood and Bayesian posterior probabilities (BPP) > 0.95 for BI were considered as significantly supported.

## 4. Conclusions

*Bougainvillea* is deemed to be one of the most popular genera in the Nyctaginaceae family, as it is used mainly as an ornamental plant. However, despite its horticultural value, its molecular and phylogenetic aspects are not well-researched. Hence, in this paper, the complete chloroplast genomes of four species (*B. glabra, B. peruviana, B. pachyphylla, B. praecox*) and one cultivar were sequenced and analyzed. In general, all *Bougainvillea* cp genomes newly sequenced from GenBank, including *Bougainvillea spectabilis*, have similar genome structures and features. They all display the typical quadripartite structure and have the same numbers of genes (131) and introns (17). Highly similar patterns were also obtained from codon usage, RNA editing, and repeat analyses. Although the *Bougainvillea* cp genomes are highly conserved and no rearrangement was observed, several highly divergent regions were identified. Moreover, phylogenetic analyses revealed the early divergence of the rarely cultivated or wild *Bougainvillea* (*B. peruviana, B. pachyphylla, B. praecox*). *B. pachyphylla* and *B. peruviana* were shown to be the basal taxa of *Bougainvillea*. Close relationships among *Bougainvillea glabra, Bougainvillea spectabilis*, and *Bougainvillea* cultivar were also confirmed. These results show that chloroplast genomes can provide sufficient information that can be used in phylogenetic studies. However, more cp genome sequences are needed to further elucidate relationships among *Bougainvillea* species and cultivars.

## Figures and Tables

**Table 1:** Complete chloroplast genome features of five *Bougainvillea* species and one cultivar.

Features	*B. glabra*	*B. peruviana*	*B. pachyphylla*	*B. praecox*	*B. cultivar*	*B. spectabilis*
Genome size (bp)	154,536	153,966	154,062	154,306	154,520	154,541
LSC length (bp)	85,708	85,159	85,181	85,474	85,688	85,694
SSC length (bp)	18,038	18,025	18,027	18,014	18,078	18,077
IR length (bp)	25,395	25,391	25,427	25,409	25,377	25,385
Total no. of genes	131	131	131	131	131	131
Protein-coding genes	86	86	86	86	86	86
rRNA	8	8	8	8	8	8
tRNA	37	37	37	37	37	37
Overall GC content (%)	36.5%	36.6%	36.5%	36.5%	36.5%	36.4%
GC content in LSC (%)	34.2%	34.3%	34.3%	34.3%	34.2%	34.2%
GC content in SSC (%)	29.5%	29.6%	29.6%	29.5%	29.5%	29.5%
GC content in IR (%)	42.8%	42.8%	42.8%	42.8%	42.8%	42.7%

**Table 2:** List of genes encoded by six *Bougainvillea* cp genomes.

Gene Category	Gene Names
**ATP Synthase**	*atp*A, *atp*B, *atp*E, *atp*F *, *atp*H, *atp*I
**NADH dehydrogenase**	*ndh*A *, *ndh*B^(X2)^ *, *ndh*C, *ndh*D, *ndh*E, *ndh*F, *ndh*G, *ndh*H, *ndh*I, *ndh*J, *ndh*K
**Cytochrome b/f complex**	*pet*A, *pet*B *, *pet*D *, *pet*G, *pet*L, *pet*N
**Photosystem I**	*psa*A, *psa*B, *psa*C, *psa*I, *psa*J
**Photosystem II**	*psb*A, *psb*B, *psb*C, *psb*D, *psb*E, *psb*F, *psb*H, *psb*I, *psb*J, *psb*K, *psb*L, *psb*M, *psb*N, *psb*T, *psb*Z
**RubisCO large subunit**	*rbc*L
**Ribosomal protein genes (large subunits)**	*rpl*2^(x2)^ *, *rpl*14, *rpl*16 *, *rpl*20, *rpl*22, *rpl*23^(x2)^, *rpl*32, *rp*l33, *rpl*36
**Ribosomal protein genes (small subunits)**	*rps*2, *rps*3, *rps*4, *rps*7^(x2)^, *rps*8, *rps*11, *rps*12^(x2) #^, *rps*14, *rps*15, *rps*16 *, *rps*18, *rps*19
**RNA Polymerase**	*rpo*A, *rpo*B, *rpo*C1 *, *rpo*C2
**Ribosomal RNA genes**	*rrn*4.5^(X2)^, *rrn*5^(X2)^, *rrn*16^(X2)^, *rrn*23^(X2)^
**Transfer RNA genes**	*trn*I-CAU^(x2)^, *trn*L-CAA^(x2)^, *trn*V-GAC^(x2)^, *trn*I-GAU^(x2)^ *, *trn*A-UGC^(x2)^ *, *trn*R-ACG^(x2)^, *trn*N-GUU^(x2)^, *trn*L-UAG, *trn*P-UGG, *trn*W-CCA, *trn*M-CAU, *trn*V-UAC *, *trn*F-GAA, *trn*L-UAA *, *trn*T-UGU, *trn*S-GGA, *trn*fM-CAU, *trn*G-GCC, trnS-UGA, *trn*T-GGU, *trn*E-UUC, *trn*Y-GUA, *trn*D-GUC, *trn*C-GCA, *trn*R-UCU, *trn*G-UCC *, *trn*S-GCU, *trn*Q-UUG, *trn*K-UUU *, *trn*H-GUG
**ATP-dependent protease**	*clp*P **
**Maturase**	*mat*K
**Hypothetical chloroplast reading frames**	*ycf*1^(x2)^, *ycf*2^(x2)^, *ycf*3 **, *ycf*4
**Acetyl-CoA carboxylase**	*acc*D
**C-type cytochrome synthesis gene**	*ccs*A
**Envelope membrane protein**	*cem*A
**Translational initiation factor**	*inf*A

Note: ^X2^ duplicated genes; * genes with one intron; ** genes with two introns; ^#^ trans-spliced gene.

## Supplementary Materials

The following are available online at https://www.mdpi.com/2223-7747/9/12/1671/s1: Table S1: Codon usage. Table S2: RNA editing sites. Table S3: List of synonymous and non-synonymous SNPs. Figure S1: Large deletions in *clp*P introns of *Bougainvillea* cp genomes. Figure S2: Large deletions in *Bougainvillea peruviana* and *Bougainvillea pachyphylla*. Figure S3: Maximum likelihood tree based on potential *Bougainvillea* barcodes.
