# Supplementary figures and images for: Comparative Analysis of Complete Chloroplast Genome Sequences of Wild and Cultivated Bougainvillea (Nyctaginaceae)
Source: Plants (Basel). 2020 Nov 28;9(12):1671. doi: 10.3390/plants9121671 (PMC7760935; doi:10.3390/plants9121671)

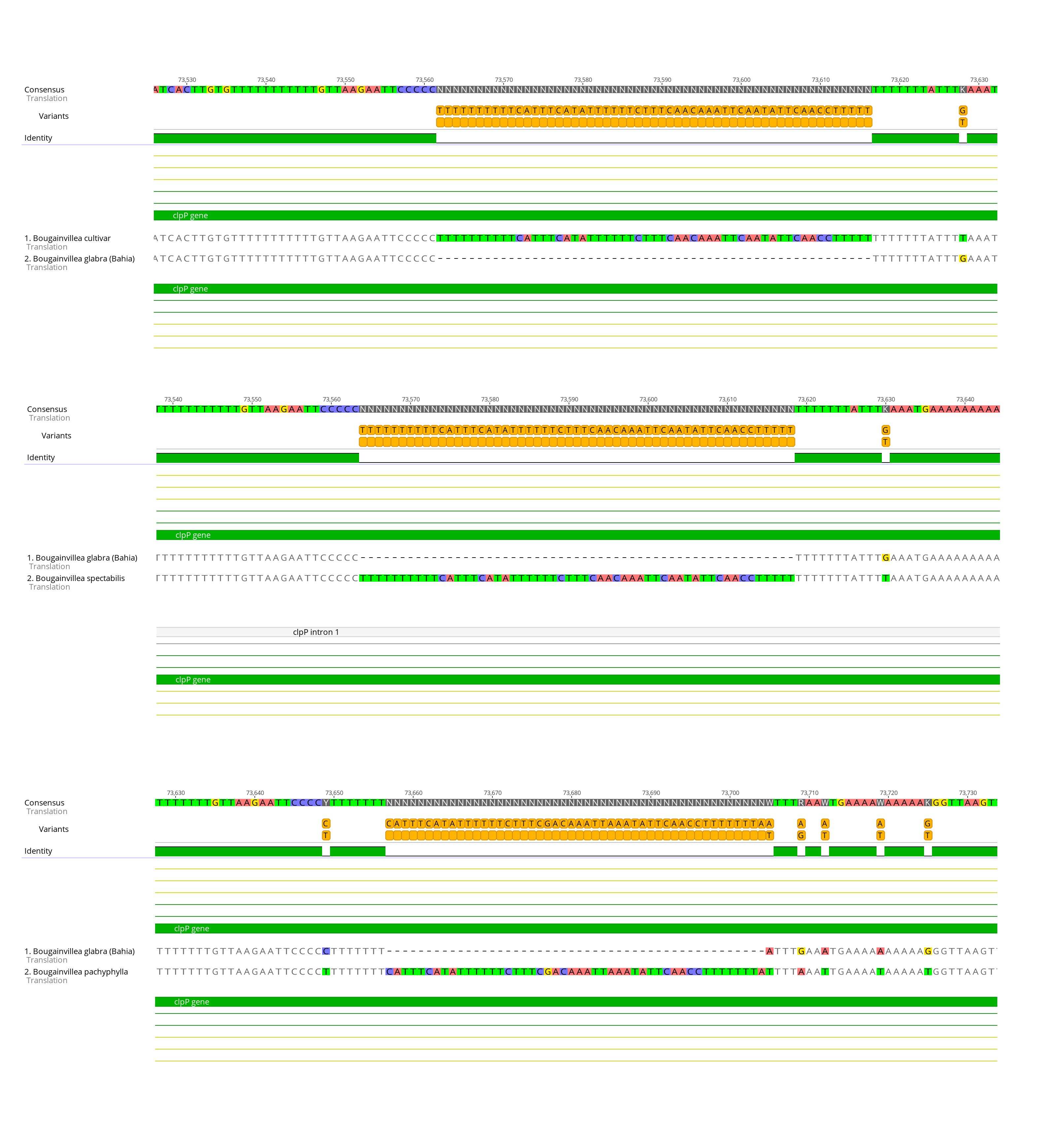

Supplement: Supplementary file 1 [file plants-09-01671-s001.zip › Supplementary Files/Figure S1 Large Deletions in clpP introns of Bougainvillea cp genomes.png]

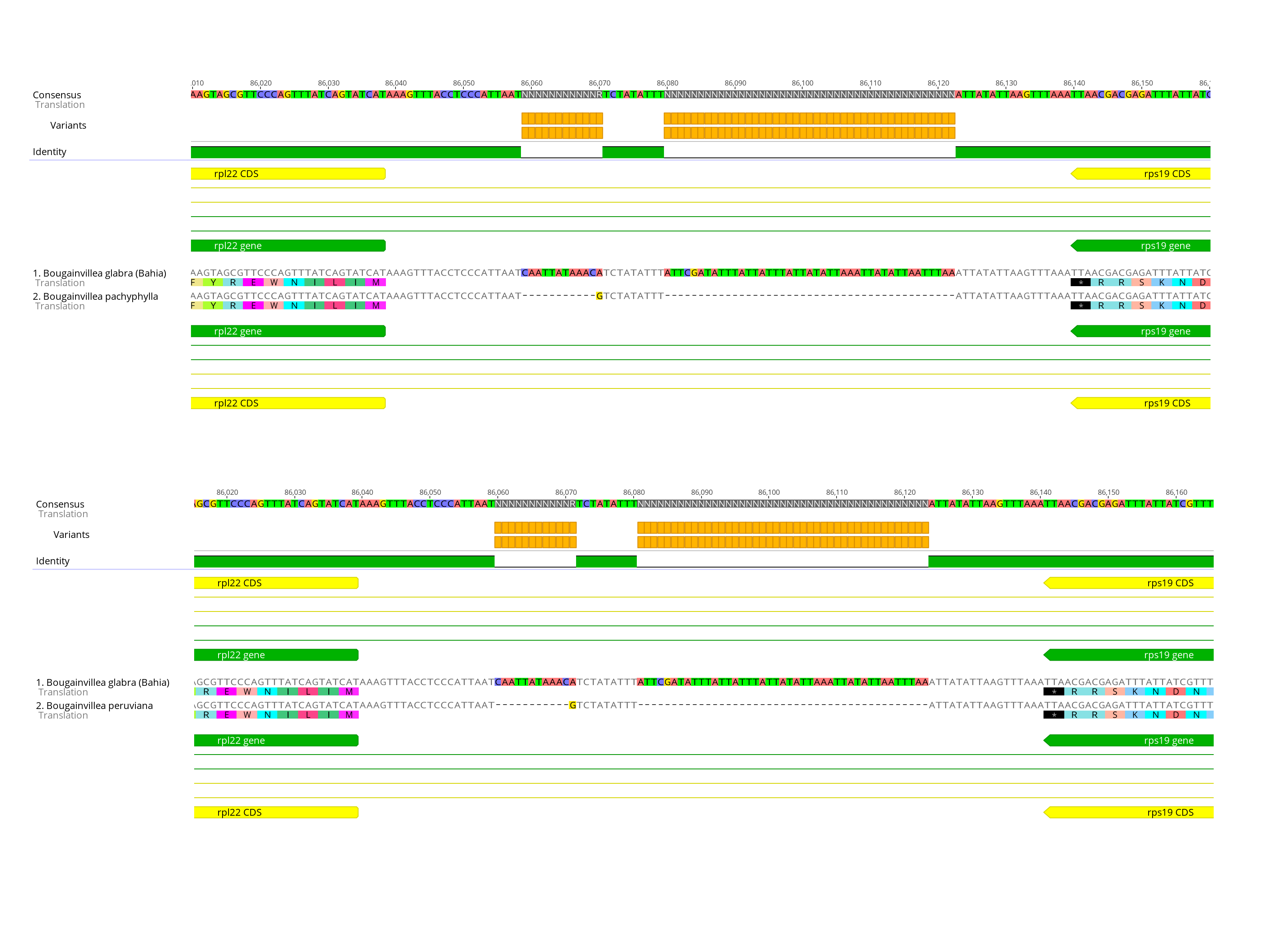

Supplement: Supplementary file 1 [file plants-09-01671-s001.zip › Supplementary Files/Figure S2 Large Deletions in B. pachyphylla and B. preuviana.png]

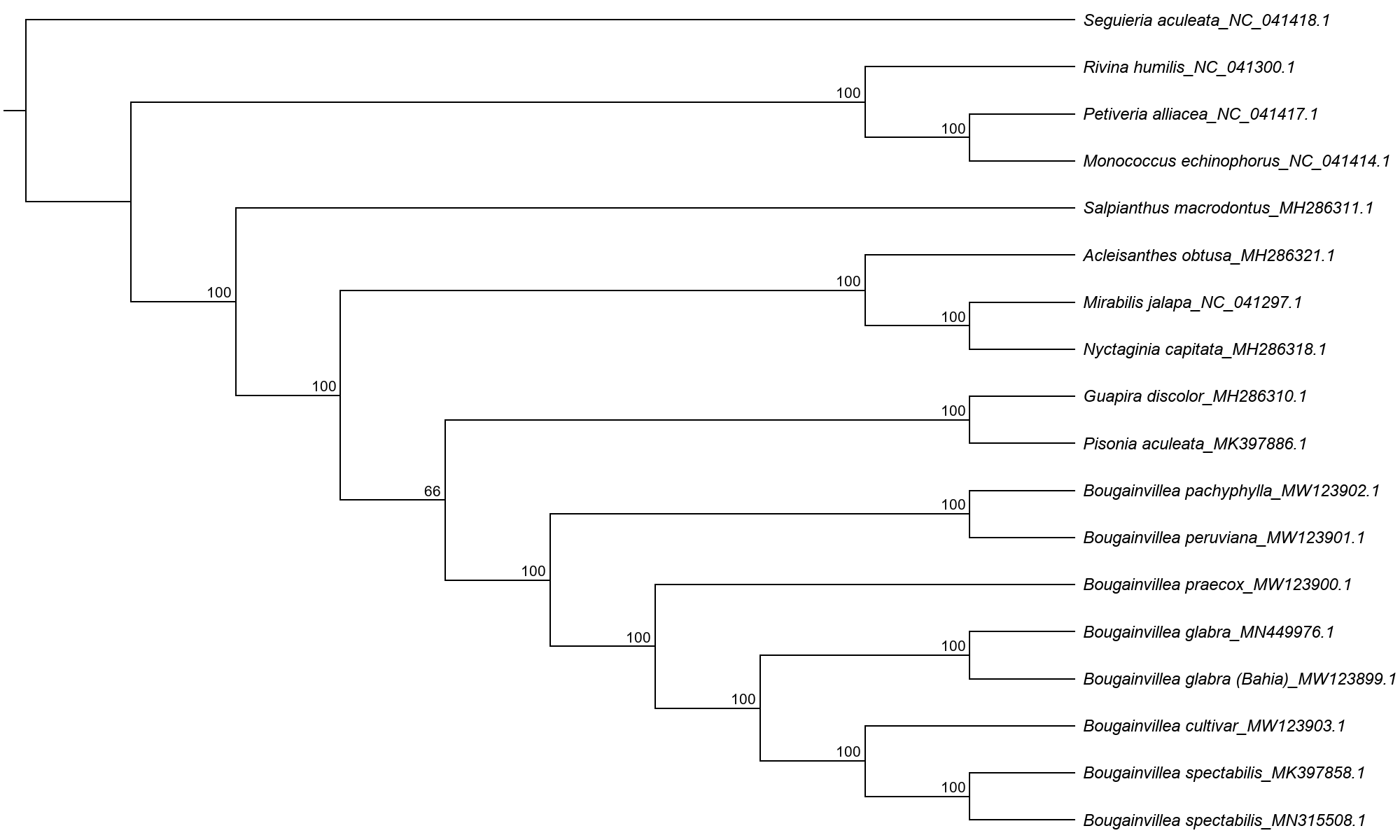

Supplement: Supplementary file 1 [file plants-09-01671-s001.zip › Supplementary Files/Figure S3 ML Tree based on Potetntial Barcodes.png]
